# Special contoured pelvic brim reconstruction titanium plate combined with trans-plate buttress screws (quadrilateral screws) for acetabular fractures with quadrilateral plate involvement through the anterior ilioinguinal approach

**DOI:** 10.3389/fsurg.2024.1438036

**Published:** 2024-09-12

**Authors:** Wei Wang, Xianhua Cai, Ximing Liu, Guodong Wang, Hui Kang, Shenglong Qian

**Affiliations:** ^1^ Department of Orthopedic Surgery, General Hospital of Central Theater Command, Wuhan, China; ^2^ Department of Orthopedic Surgery, Hubei Provincial Hospital of Traditional Chinese Medicine (Affiliated Hospital of Hubei University of Traditional Chinese Medicine), Wuhan, China; ^3^ Department of Orthopedic Surgery, South China Hospital of Shenzhen University, Shenzhen, China

**Keywords:** acetabulum, acetabular fracture fixation, quadrilateral plate, ilioinguinal approach, technique

## Abstract

**Background:**

Managing complicated acetabular fractures involving the quadrilateral plate (QLP) can be challenging for surgeons, especially when complicated by comminution and osteoporosis. Traditional implants do not provide sufficient fixed strength or a proper match. The new-type pre-contoured infrapectineal buttress plates may have drawbacks, such as inaccurate fitting on the medial surface of QLP and an inability to apply reversed compression force to resist medial displacement of femoral head. Therefore, the primary purpose of this study is to introduce a novel technique that utilizes a special contoured pelvic brim reconstruction titanium plate combined with quadrilateral screws to reduce and stabilize acetabular fractures involving the QLP through the ilioinguinal approach. Additionally, the secondary purpose is to evaluate both clinical effectiveness and radiological outcomes of this technique for QLP fractures.

**Methods:**

We conducted a retrospective analysis of prospectively collected data from 48 patients (31 males and 17 females) who suffered from acute displaced fractures of the QLP and were treated between January 2012 and December 2019 using a special contoured plate combined with quadrilateral screws. The patients' mean age was 47.56 ± 11.31 years (range: 19–73 years). Fracture patterns included 20 both-column fractures, 12 anterior column and posterior hemitransverse fractures, eight T-type fractures, five transverse fractures and three anterior column fractures with the QLP affected, all of which had femoral head protrusion. Immediate postoperative reduction quality was evaluated according to Matta's criteria. Final clinical functions were assessed during follow-up using the modified Merle d’Aubigné and Harris Hip scores (HHS).

**Results:**

The patients were followed up for an average of 48.36 ± 12.94 months (ranging from 24 to 84 months). The mean operative time was 246.08 ± 54.30 min (ranging from 178 to 397 min), and the average blood loss was 715.16 ± 263.84 ml (ranging from 400 to 2000ml). The radiological grading at postoperative stage showed anatomical reduction in 30 patients (62.50%), satisfactory reduction in 14 patients (29.17%), and poor reduction in four patients (8.33%). At the final follow-up, no re-protrusion of the femoral head was observed. In terms of functional outcome, the mean modified Merle d’Aubigné-Postel score was excellent in 26 patients (54.17%), good in 17 patients (35.42%), fair in four patients (8.33%), and poor in one patient (2.08%). The HHS was excellent in 23 patients (47.92%), good in 20 patients (41.67%), fair in four patients (8.33%), and poor in one patient (2.08%). The average HHS was 87.38 ± 7.86 (ranging from 52 to 98). Postoperative complications included lateral femoral cutaneous nerve injury in two patients, delayed wound healing and subsequent development of an inguinal hernia in one patient. Late complications were observed in two patients, with one case of heterotopic ossification and another case of post-traumatic osteoarthritis underwent hip arthroplasty within two years after surgery.

**Conclusion:**

Our results indicate that employing the contoured plate specifically designed for QLP injuries, in conjunction with quadrilateral screws through the ilioinguinal approach, can lead to positive outcomes in the treatment of displaced acetabular fractures involving the QLP. This straightforward and efficient technique offers a viable option for surgeons who are managing complex acetabular fractures.

## Introduction

Since the 1960s, unstable acetabular fractures have been treated with surgical reduction and stable fixation as proposed by Judet et al., which has become the gold standard (1). An epidemiological study based on France estimated that the incidence of acetabular fractures was 5.9/100,000/year, and both the incidence of acetabular fracture and the rate of surgical treatment were increasing speedily (2). The quadrilateral plate (QLP), which is located adjacent to the hip joint's articular surface and serving as the medial boundary of the acetabulum, has the potential to cause burst fractures and separation resulting from medial impact and femoral head thrust. Qureshi et al. (3) reported that fractures involving medial displacement of the QLP accounted for 10%–15% of all acetabular fractures. Nicol et al. (4) even reported the incidence of up to 40% in elderly patients with acetabulum fractures. For a long time, the concept and limits of QLP have been vague and controversial. Hutt et al. (5) found that 35% of acetabular fractures, particularly those involving the QLP, do not match the Letournel classification system. Even at present, the QLP is not recognized as a distinct parameter in acetabular fracture classification systems. If the acetabular fracture involving the QLP is inadequately repaired, residual incongruity may lead to post-traumatic hip arthritis and result in significant pain and dysfunction.

Over the past few decades, the optimal fixation technique or implant for complex acetabular fractures involving the QLP has remained controversial. Okelberry was the first to suggest using periarticular screws alone for fixing QLP fractures. However, this technique has proven to be challenging due to the high risks of articular or intrapelvic penetration and early loss of reduction. Although with modern technologies such as three dimensional (3D) navigation systems, the risk of joint penetration can be reduced (6), in cases of comminution and osteoporosis, the utilization of screws alone is insufficient for fixing QLP fractures. Letournel (7) and Matta (8) described the classic ilioinguinal approach for addressing acetabular fractures by directly exposing the anterior column and QLP. This approach involves using a pelvic iliopectineal brim reconstruction plate, with screws placed parallel to the QLP. The procedure requires the insertion of periarticular long screws in close proximity to the fracture and through the QLP. This technique was previously considered the standard surgical treatment for displaced QLP fractures.

To prevent the penetration of screws into the hip joint when using the traditional fixation method of lag screws over a suprapectineal plate, some surgeons propose using extraosseous fixation, such as bone surface buttress plates, for repairing displaced fractures of the QLP. Some scholars have recommended using an appropriately contoured spring plate positioned beneath a pelvic brim reconstruction plate to reinforce QLP fractures (9). Although this technique was found to be effective in biomechanical studies and gained widespread acceptance (10), accurately contouring the spring plate to match the medial wall was also challenging. Additionally, the fulcrum of the spring plate was relatively single, and the fixation range was small, resulting in the ability of this buttress plate to resist of the rotation of the fracture fragments is poor. In recent years, several novel types of pre-contoured buttress plate have been designed and reported to treat acetabular fractures involving the QLP (Table 1) (11–19), some of which have been applied in clinical treatment and have achieved good clinical efficacy. However, because of irregular shape of the QLP and the inward displacing forces from femoral head, these new implants cannot completely match the medial surface of QLP and provides sufficient fixtion strength. Therefore, there is still a risk of re-displacement of QLP fracture fragments. Additionally, due to the limited number of clinical application cases and low popularity, these new pre*-*contoured quadrilateral surface reinforcement plates have not been mass-produced. Therefore, there is an urgent need to design and develop a new internal fixation technique and strategy that is simple, easy to operate, and can improve treatment without requiring special device.

**Table 1:** The comparison of different novel pre-contoured implants in recent years.

Literature	Novel implant	Patients (*n*)	Approach	Reduction outcome (Matta's radiological criteria) (*n*)	Follow-up	Complications (*n*)
Schäffler A, et al. (11)	The acetabular anatomical wing plate	8	The modified Stoppa approach and the first window of the ilioinguinal approach	Anatomic: 8	3 months	No reported
Taller S, et al. (12)	The Omega plate	15	The modified Stoppa approach	Anatomical and Imperfect: 12 Poor: 3	8.5 months in 11 patients; 4 were shortly after surgery	Post-injury arthritis at six months: 1
Zhang R, et al. (13)	The anatomic quadrilateral surface plate	26	The modified Stoppa approach	Anatomic: 23 Imperfect: 3	mean 28.81 months	Obturator nerve injury: 2 Corona mortis rupture: 1
Zha GC, et al. (14)	The acetabular fracture reduction internal fixator	24	The Ilioinguinal approach	Anatomical: 17 Imperfect: 5 Poor: 2	Mean 45.7 months	Osteoarthritis: 4 Total hip arthroplasty: 2
Sen RK, et al. (15)	The anatomic quadrilateral plate	33	The Stoppa approach or Modified Ileo-femoral’ approach	Anatomical: 28 Imperfect: 5	Minimum 1 year	Total hip arthroplasty (within six months): 1 Had an issue with mobility: 3 Minor limitations in usual activities: 6
Wang C, et al. (16)	The three- dimensional printed patient-specific Ti-6Al-4 V plates	15	The lateral-rectus abdominis approach	Anatomical: 10 Imperfect: 4 Poor:1	At least 1 year	Loosening of a pubic screw: 1
Ciolli et al. (17)	the suprapectineal quadrilateral surface plates	34	The anterior intrapelvic approach	Anatomical: 26 Imperfect: 7 Poor:1	Mean 20.7 months	Deep infection: 1 Screw penetration into the joint: 1 Deep venous thrombosis: 3
Wan Y, et al. (18)	The novel infrapectineal buttress plates	23	The Ilioinguinal approach	Anatomical: 14 Imperfect: 5 Poor: 4	At least 1 year	Lateral femoral Cutaneous nerve injury: 3 Heterotopic ossification: 1 Slight hip pain: 4
Kyung KD, et al. (19)	The anatomical suprapectineal quadrilateral surface plate	16	The modified Stoppa approach	Anatomical: 14 Imperfect: 2	At least 1 year	Post-traumatic arthritis and subsequent total hip arthroplasty: 2

*n*, number of patient.

In this study, we introduce a new technique that uses screws to reduce and stabilize bone surfaces, preventing medial displacement of the acetabular fractures involving the QLP. This technique is based on the concept of pelvic brim reconstruction plates and screws composite. Considering the anatomical and biomechanical features of the acetabulum, we emphasize that the reconstruction plate needs to be contoured specially. This ensures that the original periarticular intraosseous screws are transferred to the medial surface of QLP, where they are distributed like a raft (called quadrilateral screws) to buttress the medial wall. As a result, the risk of articular penetration is eliminated, and the stability of the implant is maintained. We have been utilizing this technique for over ten years and have found it to be safe, straightforward, and effective in achieving stable fixation in cases with medial displacement of the acetabular fractures involving QLP. It is particularly worth mentioning that by adopting this technique, most surgeons can effectively repair complex acetabular fracture involving the QLP using ordinary reconstruction plates and screws with the available tools, even in the absence of special preformed buttress plates.

The primary purpose of this study is to provide a detailed description of the surgical technique mentioned above. Our secondary purpose is to evaluate the clinical outcomes and effectiveness of treating complicated acetabular fractures involving the QLP using a single ilioinguinal approach, based on our team's experience.

## Materials and method

### Patients

This study was approved by the ethics department of General Hospital of Central Theater Command [(2023)002-02]. Although this was a retrospective and observational study with medical records, we also followed the Declaration of Helsinki and relevant policies in China, we have consensus with all participants, and all patients signed informed consent.

From January 2012 to December 2019, our trauma center (level Ⅰ trauma center) has treated 348 patients with acetabular fractures using open reduction and internal fixation (ORIF). We included individuals aged between 18 and 75 years with acetabular fractures involving the QLP, treated with special contoured pelvic brim reconstruction titanium plate combined with trans-plate buttress screws (quadrilateral screws) through the anterior ilioinguinal approach, and a follow-up more than 18 months. We excluded patients who were under 18 years or over 75 years old, simple fractures of anterior wall, those with fractures primarily involving the posterior wall or complicated posterior column fractures requiring a posterior approach, those with the surgery was delayed beyond three weeks, those with pre-existing arthrosis and femoral head avascular necrosis, those with less than 18 months of follow-up, and inadequate quality of follow-up.

**Figure 1:**
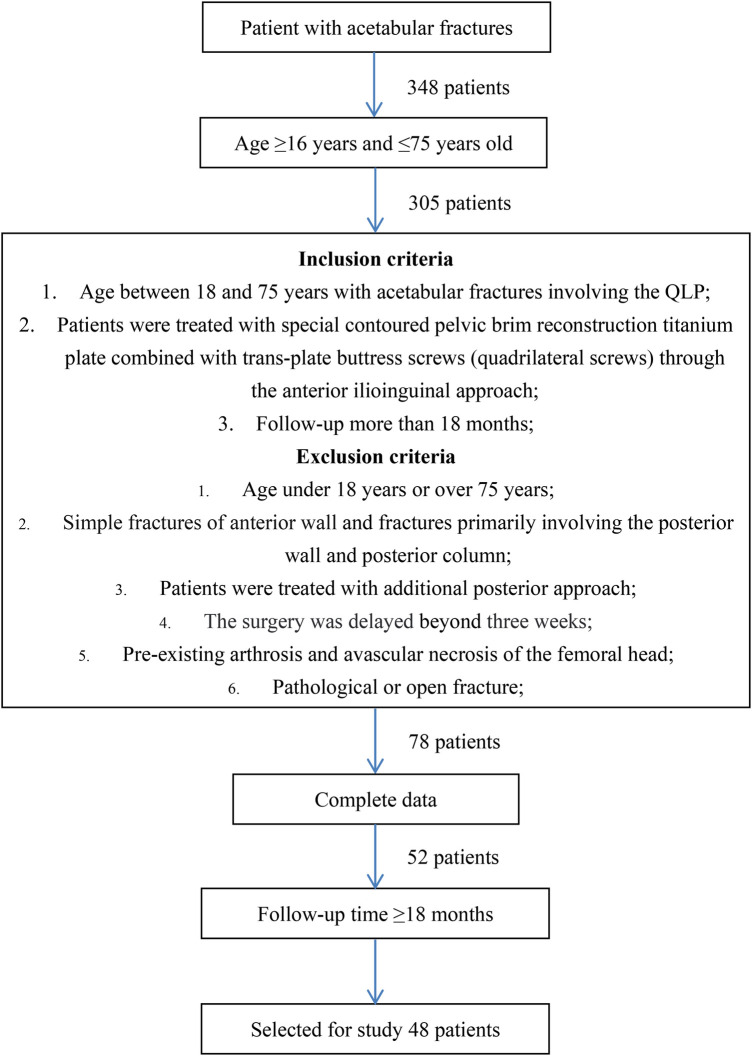
Study flow diagram.

The technique described in this paper was used to treat 52 patients with the acetabular fractures involving QLP through a single ilioinguinal approach. After six months of fracture healing, four patients were lost during the follow-up period, resulting in a study population of 48 patients (Figure 1), 31 male and 17 female, and the mean age was 47.56 ± 11.31 years (range: 19–73 years). The fracture patterns included 20 both-column fractures, 12 anterior column and posterior hemi-transverse fractures, eight T-type fractures, five transverse fractures and three anterior column fractures with the QLP affected. Patients with other concomitant injuries included 3 cases of visceral injuries (1 bladder injury, 1 abdominal injury, 1 thoracic injury), 4 cases of upper limb fractures (2 ulna ocleranons, 1 ulna and radius, 1 distal radius), 6 lower limb fractures (2 femurs, 1 tibia, 2 ankles, 1 calcaneus), 2 cases of craniocerebral injuries and 2 cases of spinal fractures. The mean surgical delay was 8.10 ± 2.75 days (range: 3–17 days).

### Preliminary biomechanical studies

We have previously conducted a series of biomechanical evaluations to confirm the stability of our technique, which were obtained through various tests, including finite element analysis. For example, compared with the fixation systems of double–column plates and anterior column plate combined with posterior column screws, the anterior column special contoured plate combined with quadrilateral screws provided reliable biomechanical properties for T-shaped fractures and anterior column and posterior hemi-transverse fractures (20, 21). Furthermore, according to the mechanical force transmission characteristics of the pelvic ring, a subsequent biomechanical study based on finite element analysis showed that recovery of the integrity of the pelvic ring was the prerequisite for the quadrilateral screws fixation systems providing sufficient biomechanical stability in treating acetabular double–column fractures (22). These studies served as the foundation for the clinical application of our technology.

## Surgical technique

### Surgical position and approach

Following the induction of general anesthesia, the patient was positioned supine or floating supine (the affected side above the waist and buttock was raised slightly, while the externally rotated ipsilateral lower limb was flexed slightly in order to relax the iliopsoas and the neurovascular bundle). As described by Letournel (7), we used the classic ilioinguinal approach, to access the injured acetabulum. The three standard surgical windows exposed the regions of the anterior column, anterior sacroiliac joint, pelvic boundary, and quadrilateral lamina. Simultaneously, the sacrotuberous ligament and sacrospinous ligament were preserved.

### Reduction and temporary fixation

After debriding the broken fragments and hematomas, as a general rule, the medial dislocated femoral head was reduced and placed in an anatomic position beneath the lateral dome. An unobstructed reduction was allowed, and the femoral head can be severed as a template for the surgical reduction of the acetabular dome fragment and the articular surface.

The proximal end of the fracture serves as a marker, and the sequence for reducing the fracture starts from the periphery to the central and from proximal to distal. When the fracture involves both columns, the anterior column was reduced first, under direct vision according to the level of fracture line of the ilial wing and iliopectineal brim. The residually displaced posterior column and the quadrilateral surface can be reduced slowly using a ball-spiked pushers or pelvic reduction clamps.

If the fracture is accompanied by acetabular dome impaction, the impacted dome will be reduced under direct visualization through the fracture gap, as described by Laflamme and Hebert-Davies (23), to deteriorate the displacement of the medialized quadrilateral fragment. Occasionally, osteotomy using an iliac cortical window adjacent to the joint, as described by Casstevens et al. (24), can be helpful. When the fractures involve the iliac wing or affect the stability of the pelvic ring, achieving anatomical reduction and fixation with a plate takes priority. For posterior column fractures, two posterior column lag screws were inserted from the pelvic brim towards the posterior column. Finally, the continuity of the acetabular fracture was repaired, and Kirschner wires or plates were used to maintain temporary reduction (25).

### The technique for contouring the reconstruction plate

**Figure 2:**
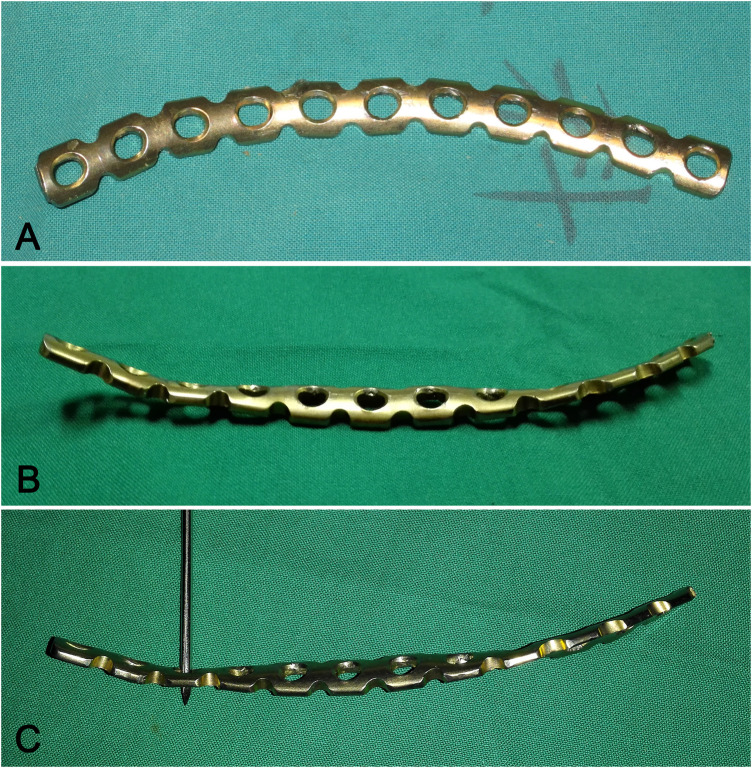
**(A)** A 3.5 mm pelvic brim reconstruction plate (supero-inferior views). **(B)** A specially-contoured pelvic brim reconstruction plate with both ends of the plate warping upwarp slightly and the middle segment slightly inclined to the true pelvic (supero-inferior view). **(C)** A specially-contoured pelvic brim reconstruction plate. (lateral view).

After reducing the fragments, plate fixation was applied to achieve stabilization. Prior to fixing the QLP fractures, a 3.5-mm usual pelvic brim reconstruction plate (Synthes, Switzerland) was specially contoured before fixing the fractures involving QLP (Figure 2). The plate length was determined by the anterolateral of the sacroiliac joint along the upper edge of the QLP to the superior aspect of the ramus and tuberculum pubicum. The reconstruction plate is contoured in a special manner as follows. First, the concave side arc of the curved pelvic brim plate faces the surgeon, and then the middle segment of the reconstruction plate is fixed using a plate bender. Next, both sides of the reconstruction plate were reversed twisted from inside to outside with the other plate bender respectively. As a result, both ends of the plate were slightly upturned, and the plane of the middle segment of the plate appeared as an oblique plane with lower inner side and a higher outer side. Additionally, the slope of the inclined plane (approximately 14**–**16 degrees) was slightly steeper than that shown with the pelvic brim above the QLP. In contrast, the plane of both plate ends presented an oblique plane with inner high and outer low. The contoured plate did not fit the pelvic brim region above the true pelvis, and both ends of the plate slightly warped upwards, while the plane of the middle segment of plate inclined slightly towards the true pelvis. (The process is demonstrated in Supplementary File 1).

### The placement technique of specialized contoured reconstruction plate

**Figure 3:**
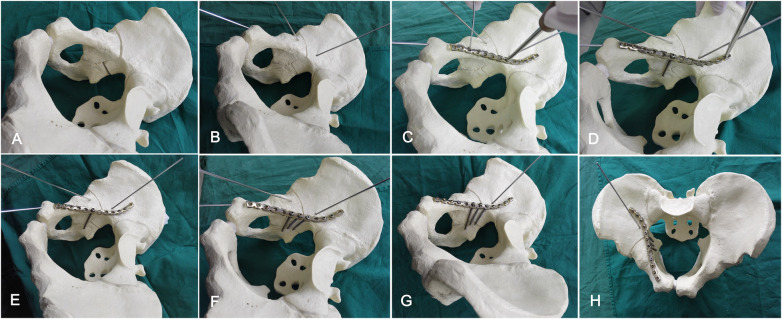
Bone model depicted our surgical technique for fixing anterior column and posterior hemitransverse fractures. **(A)** Bone model with displaced anterior column and posterior hemitransverse fractures. **(B)** The first step involved reduction of the displaced QLP fracture into its proper position and temporary fixation with Kirschner wires. **(C)** The special contoured reconstruction plate was placed along the pelvic brim. The middle segment of plate was moved slightly and partially protruded into the ture pelvics with the 1/3–1/2 width of the plate plane. **(D)** The first screw was placed in the superior aspect of superior pubis ramus, the second screw, known as the “quadrilateral screw”, was inserted closely through one middle segment hole of the plate in order to prevent medial displacement of the QLP fracture components. **(E)** It was secured posteriorly using at least two cortical screws (3.5 mm) fixed each end of the plate holes to capture the dense uninjured bone and stabilize the anterior column. In this process of nailing*,* the special contoured plate was gradually compressed and occurring elastic deformation to fit the pelvic brim. **(F)** Drilling holes or groove though the middle segment of the plate above the quadrilateral and along the surface of the QLP. In this process, the drill bit should be prevented from entering the bone completely. The others quadrilateral screws were inserted through the holes or groove alone the pelvic brim and parallel to the quadrilateral surface, the screws partially (the 1/3–1/2 transverse diameter) entering the bone lamella of QLP but without entering the joint. **(G,H)** After the others screws were placed, the stability of the fracture was obtained, and the temporary Kirschner wires were removed (the remaining Kirschner wires simulate the posterior column lag screw). These quadrilateral screws were distributed like a raft construct on the medial surface to buttress the medial wall. **(G)** The pelvis lateral view. **(H)** The pelvic entrance view.

The specialized contoured plate was placed along the pelvic brim (Figures 3A–C). Based on the anatomical structure of the pelvic ring and the plate's placement position, the structure was divided into three regions: the iliac, quadrilateral, and pubic regions. The proximal end of the plate arrives at the anterolateral ilium and the anterior sacroiliac joint (the iliac region), while the distal end of the plate reaches the upper surface of the superior ramus (the pubic region). The middle segment of the contoured plate covers the upper edge of the QLP (the quadrilateral region). It's worth noting that this segment needed to be moved slightly and partially protruded into the true pelvis, extending beyond the pelvic boundary with the 1/3–1/2 width of the plate plane. Because the middle segment of the contoured plate has a slightly steeper slope than the pelvic brim above the QLP, the plane of the quadrilateral region inclines towards the true pelvis, causing both ends of the contoured plate warp upward slightly along the pelvic brim. (The process is demonstrated in Supplementary File 1).

### The placement technique and sequence of screws

Firstly, the first screw (3.5 mm, Synthes, Switzerland) was inserted in the superior ramus of the pubis. Then, through one quadrilateral region hole of the contoured plate, drilling or slotting close to the pelvic brim and parallel down the inner surface of the QLP, simultaneously, the drill bit was prevented from fully penetrating the bone lamella during this process. The second screw, known as the “quadrilateral screw”, was inserted through the slot and placed closely against the medial surface of QLP to prevent medial displacement of the quadrilateral fracture fragments (Figure 3D). After that, at least two cortical screws were inserted at each end of the plate holes in order to capture the dense uninjured bone and stabilize the anterior column (Figure 3E).

Once preliminary fixation was achieved, drilling or slotting along the pelvic brim and parallel to the medial surface of the QLP as described previously, the remaining 1–3 quadrilateral screws were sequentially inserted close to the pelvic brim and parallel down the quadrilateral surface through the residual holes on the quadrilateral region of the contoured plate (Figure 3F). These screws were inserted partially (around 1/3–1/2 of the transverse diameter) into the quadrilateral osseous lamella without penetrating the joint, ideally exceeding the fracture line of the quadrilateral surface at approximately 10 mm in length. As a result, these quadrilateral screws passed through the plate holes and captured the pelvic brim, creating a raft construct on the medial surface of the QLP to buttress the medial wall. Finally, the remaining screws were inserted and tightened through the residual plate holes (Figures 3G,H). (The process of placing quadrilateral screws as shown in the animation in Supplementary File 2).

During the nailing process, the specially contoured plate was compressed progressively, the shape of the plate was altered because of torsion and elastic recoil. By elastic deformation of the plate, it provided a strong clamping force for the quadrilateral screws. This technique has two main benefits: firstly, the plate abuts against and fits the pelvic brim; secondly, the quadrilateral screws exert persistent outwardly-directed pressure on the medial wall, which preventing medial displacement of the QLP fracture fragments. This indirect fixation technique provides sufficient elastic buttressing and stability to reduce the QLP fracture fragments. Furthermore, the buttressed region on the medial surface could be extended by adjusting the number, distribution, and length of the quadrilateral screws in order to accommodate comminuted and osteoporotic fractures of the QLP.

**Figure 4:**
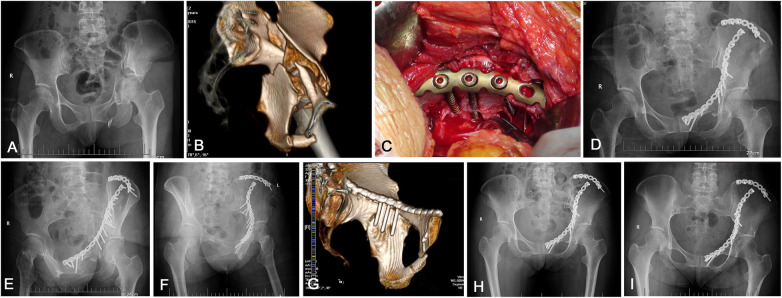
An example showing the special contoured pelvic brim reconstruction titanium plate combined with the quadrilateral screws technique was used for a complicated acetabular fracture involving the QLP. **(A)** Preoperative AP x-ray of the pelvis shows a displaced both-column acetabular fracture pattern with protrusion following a traffic accident in a 38-year-old female. **(B)** Preoperative computer tomography (CT) scan with 3D-multiplanar reconstruction. **(C)** Intraoperative clinical photograph showing reduction and fixation was completed, three quadrilateral screws were placed through the middle segment holes of the contoured plate above the QLP, the screws partially (the 1/3–1/2 transverse diameter) entering the medial bone lamella of QLP. **(D–F)** Postoperative AP, obturator oblique and iliac oblique views demonstrating the fracture was anatomically reduced and stable. **(G)** Postoperative CT scan with 3D reconstruction demonstrating three quadrilateral screws were placed on the surface of medial wall, it were distributed as a raft construct, the strong holding force was provided by the special pre-contoured plate to resist the medial displacement of QLP. **(H)** After 3 months, the fracture had completely united and the reduction was maintained. **(I)** The AP view after 3 years showing a good radiological result with no evidence of osteoarthritic change.

**Figure 5:**
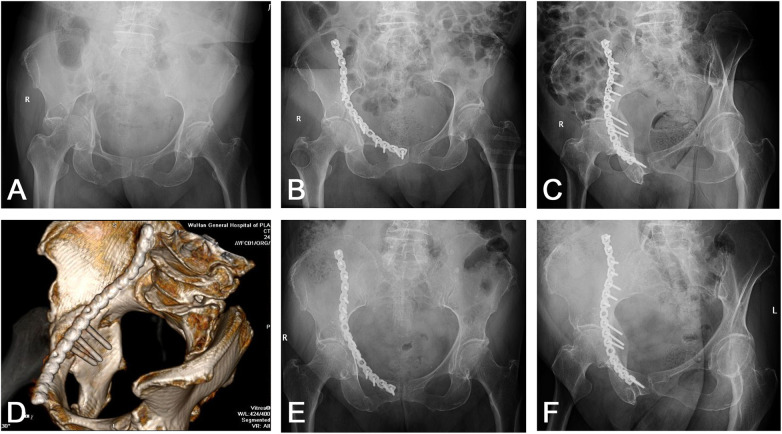
Case of a 67-year-old woman had a transverse acetabular fracture and superior pubic ramus fractures. **(A)** Preoperative AP view x-ray shows a protrusion of the femoral head. **(B)** Postoperative AP and **(C)** iliac oblique view radiograph shows a satisfactory reduction of the QLP fractures. **(D)** Postoperative CT scan with 3D reconstruction demonstrating three quadrilateral screws were placed parallel to the quadrilateral surface. **(E,F)** Postoperative AP and iliac oblique views x-rays of the pelvis at 4 years after the operation showing an excellent radiological outcome, and the patient was symptom free.

Once the fixation was completed, satisfactory reduction and stability were verified using a C-arm. Each screw was also checked to ensure that it did not penetrate the joint. After placing a suction drain in the deep and shallow layers of the iliac fossa extending to the posterior region of the pubic, the wound was irrigated and repaired in layers. (Typical cases are shown in Figures 4, 5).

### Postoperative management

Prophylactic antibiotics were administered for 24–72 h after surgery, and the drainage tube was removed within 72 h if the drainage flow was less than 20 ml in a 24-hour period. For postoperative pain management, all patients were administered the multimodal postoperative pain management protocol, which included both intravenous and oral pain medications. The postoperative rehabilitation program commenced immediately after the patient regained consciousness following anesthesia and persisted throughout the recovery period. The rehabilitation program was divided into two phases. The first phase, lasting 4–6 weeks, focuses on strengthening the muscles of the unilateral front thigh/lateral hip, and using passive and active strengthening training in a non-weight bearing manner. The second phase, lasting 6–12 weeks, was a medium-term partial weight-bearing period that involved multi-joint motion exercises and gait training. After completing this 12 weeks period, patients were encouraged to progress towards engaging in exercises with full weight-bearing, increasing the strength by 25% each week. Thromboprophylaxis was administered continuously for a duration of six weeks.

The immediate postoperative radiographs, which included standard antero-posterior (AP) view and Judet views x-rays, were evaluated according to the criteria described by Matta (26). The acetabular reduction quality was classified as anatomical, satisfactory, or poor. Clinical function assessment at the final follow-up included the modified Merle d'Aubigné score and the Harris Hip Score (HHS) (27). Postoperative follow-up included clinical and radiographic examinations were performed at 1, 3, 6, 12months after treatment, and then every year afterward. Furthermore, all perioperative complications were documented. The final follow-up and evaluation were conducted by two independent orthopaedic surgeons who did not participated in the patient's definitive care or surgery.

## Results

Surgical outcomes revealed a mean operative time of 246.08 ± 54.30 min (range: 178–397 min) and an average blood loss of 715.16 ± 263.84 ml (range: 400–2000ml). The study with an average follow-up time of 48.36 ± 12.94 months (range: 24–84 months) after surgery. All patients exhibited radiological evidence of fracture healing within five months after surgery. The quality of reduction was evaluated in all patients using Matta radiological criteria, anatomical reduction observed in 30 patients (62.50%), satisfactory reduction in 14 patients (29.17%), and poor reduction in four patients (8.33%) (Table 2).

**Table 2:** Patient demographics.

Variable	*n* (48)
General data	
Age (years)	47.56 ± 11.31 (range: 19–73)
Gender: male/female	31/17
Operation time (min)	246.08 ± 54.30 (range: 178–397)
Blood loss (ml)	715.16 ± 263.84 (range: 400–2,000)
Time to operation (days)	8.10 ± 2.75 (range: 3–17)
Mechanism of injury	
Falling	20 (41.67%)
Traffic accident	25 (52.08%)
Crashing	3 (6.25%)
Concomitant injuries	
Head injury	2 (4.17%)
Visceral injury	3 (6.25%)
Upper limb fractures	4 (8.33%)
Lower limb fractures	6 (12.50%)
Spine fracture	2 (4.17%)
Letournel classification	
T-shaped fracture	8 (16.67%)
Both column fracture	20 (41.67%)
Anterior and posterior hemi-transverse	12 (25%)
Tranverse fracture	5 (10.42%)
Anterior column	3 (6.25%)
Follow-up time(months)	48.36 ± 12.94 (range: 24–84)
Matta radiological outcome	
Anatomical (<1 mm)	30 (62.50%)
Satisfactory (2–3 mm)	14 (29.17%)
Poor (>3 mm)	4 (8.33%)
Complications	
Vascular injury	1 (2.08%)
Lateral femoral cutaneous nerve injury	2 (4.17%)
Inguinal hernia	1 (2.08%)
Heterotopic ossification	1 (2.08%)
Posttraumatic arthritis	1(2.08%)

*n*, number of patient.

At the final follow-up, 26 patients (54.17%) achieved excellent functional scores on the modification d'Aubigne and Postel scale, 17 (35.42%) had good scores, four (8.33%) had fair scores, and one (2.08%) had a poor score. The HHS was excellent in 23 patients (47.92%), good in 20 (41.66%), fair in four (8.33%), and poor in one (2.08%). The average score was 87.38 ± 7.86 (range: 52–98). During the operation, three patients (6.25%) experienced intraoperative complications, including bleeding caused by injury to the corona mortis in one patient, and lateral femoral cutaneous nerve injury in two patients. All nerve injuries resolved within six months after surgery. One patient (2.08%) experienced early complications following surgery, with delayed wound healing and the subsequent development of an inguinal hernia. Late complications were observed in two patients (4.17%), including one case of Brooker type I heterotopic ossification and another instance of posttraumatic osteoarthritis necessitating total hip arthroplasty within two years of the initial surgery. (Relevant clinical data supporting the findings of this study as shown in Supplementary File 3).

## Discussion

Management of acetabular fractures involving the QLP remains a technical challenge. Specialists worldwide have proposed various methods and implants to address this pathology. In the early period, to avoid the risk of periarticular long screws mistakenly entering the hip when placed parallel to the QLP through the pelvic brim plate, conventional fixation techniques often left the holes of the reconstruction plate above the QLP being vacant, resulting in a loss of direct fixation. Mears (28) first described the “buttress plate” technique. Subsequently, many studies reported the use of various reduction techniques with buttress plates to address comminuted fractures of the QLP. These techniques including the traditional spring plate (such as T-shaped, L-shaped, or one-third tubular plate) and the infrapectineal border buttress plate. Because of the fulcrum of these buttress plates was relatively single, and the fixation range was small, multiple clinical and biomechanical studies have indicated that the stability and strength of these plates in treating QLP fractures of the acetabulum are inadequate (9, 10, 29). In recent years, various novel designed pre-contoured anatomical plates with quadrilateral surface reinforcement have been developed and have become partially commercially available (Table 1). These implants are specifically designed to span both columns, fit the complex anatomical structure of the area, and prevent medial displacement of the QLP by combining with an infrapectineal buttress plate.

However, these new pre-contoured implants are merely modifications of classical techniques and have several deficiencies. Firstly, although the anatomical infrapectineal buttress plate fixation has been used to prevent displacement of the QLP fractures, it is not suitable for fixing fractures involving the greater sciatic notch, comminuted fractures, osteoporotic fractures of the QLP, or low transverse acetabular fractures (30, 31). Secondly, the potential “safe zone” on the QLP for secure infrapectineal plate-screw placement was limited (32). Thirdly, due to the irregular morphology of the medial surface of the QLP, achieving a precise fit of these stiffer pre-contoured buttress plate can be challenging, leaded these novel infrapectineal buttress plates were diffcult to be contoured accurately to fit the QLP concavity, and unable to exert compressive forces on the quadrilateral surface. Even the stability of the fracture fragments may be affected or induced micro movement by the deformation force generated when tightening the screw. Furthermore, the quadrilateral surface buttress plate merely plays a static blocking role or helps maintain the reduction of quadrilateral fracture fragments, the deforming force of the femoral head may cause a slight separation or intrapelvic re-displacement of the medial wall, leading to hip joint incongruity or loss of reduction. Ultimately, the stability of the fracture was reducing.

Alfonso et al.'s (33) biomechanical studies have also confirmed above-mentioned view. They found that compared to traditional fixation techniques with reconstruction plates and screws, the use of the pre-contoured acetabular buttress plates alone will lead to malreduction and increased risk of redisplacement in fixation of a transverse acetabular fracture model. It is worth mentioning that, compared to classical fixation techniques, the aforesaid novel pre-contoured implants have only been applied in a few patients and their effectiveness has not been sufficiently verified. The 3D printing plate technology is currently in the development stage and it comes with high costs. Therefore, these findings suggest the need to develop new techniques and strategies for improving the treatment of acetabular fractures involving the QLP.

The advantages of our technology are reflected in these aspects: (1) The risk of screw penetration into the joint is significantly reduced. Several biomechanical studies have indicated that the conventional suprapectineal reconstruction plate with periarticular long screws through the QLP is the most effective configuration for preventing medial re-displacement of the QLP (29, 34, 35). However, there is a higher risk of screw penetration into the joint. Even Ciolli et al. (17) reported one case of postoperative complications with screw penetration into the joint when using suprapectineal quadrilateral surface plates to fix acetabular fractures involving the anterior column. In this study, we describe a technique that addresses this issue by shifting the primary periarticular intraosseous screws to the medial surface of the QLP. This method reduces the risk of joint penetration and creates a raft-like structure that buttresses the medial wall, providing direct fixation for the QLP. It is important to note that the quadrilateral screws are inserted safely on the medial wall surface under direct vision, without the risk of penetration the joint, thus minimizing radiation exposure for both patients and surgeons. (2) The quadrilateral screws can accurately match the medial surface of QLP and provide potential dynamic compression and buttress force against the protrusion of the femoral head into the pelvis. Our technique involves carefully contouring the pelvic brim reconstruction plate, then the screws on both sides of the plate were tightened, the specific contoured plate toke place elastic deformation and provided a persistent holding force for the quadrilateral screws. This process creates a more accurate fit to the medial wall for these screws, and provides multi-point elastic fixation for fractures of the QLP. As a consequence, the potential compression and buttress force provided by the quadrilateral screws on the medial surface of the QLP is neutralized by the force of the secondary medial displacement of the femoral head. (3) The buttress range of the QLP can be dynamically adjusted according to fracture patterns. It is worth mentioning that our technique can also expand the buttressed region on the medial wall surface, according to varies of distribution and length of the quadrilateral screws, for comminuted and osteoporotic fractures. (4) Our technology is simple, safe, and easy to operate. It can improve the treatment of acetabular fractures involving the QLP without requiring a special device.

The technique we developed was applied to treat 48 patients with acetabular fractures involving QLP disruption. We achieved anatomical and satisfactory reduction in 91.67%（44/48）of the cases. Furthermore, none of our patients experienced any redisplacement or implant-related complications during a mean 48 months of follow-up. A retrospective series by Patterson et al. (36), reported that the hip survival rate of acetabular fractures after ORIF within 2 years was 95%, and the reoperation rate with total hip arthroplasty was 2.50%. For elderly patients with acetabular fractures, Smakaj et al. (37) reported that the incidence rate of secondary osteoarthritis and subsequent total hip arthroplasty was up to 29.2%（7/24）with ORIF treatment for acetabular fractures. They believe that the “combined hip procedure” is the optimal treatment option for elderly patients with poor expected outcomes in terms of joint survival. In our study, the hip survival rate was slightly higher, only one patient (2.08%) underwent total hip arthroplasty within 2 years after the primary acetabular fracture operation. Compared to others similar studies, on the one hand, the average age of our acetabular fracture patients was lower, and fractures with posterior wall comminution or preexisting arthrosis was excluded, on the other hand, because of the clinical hip function has a significant relationship with the postoperative reduction quality of acetabular fractures (17, 26, 38). We achieved a higher rate of anatomical and satisfactory reduction for QLP fractures, a lower rate of reduction loss and complications, as well as a higher rate of hip survival within 2 years. This may be attributed to the elastic fixation provided by the quadrilateral screws for QLP fractures. Our results suggest that the technique is a safe and effective internal fixation strategy for acetabular fractures involving the QLP.

Fracture patterns involving the QLP are rather complex. According to Matta and Merritt’ (39), one surgical approach should be used to address acetabular fractures if possible. They disapproved of the use of two incisions simultaneously or successively. Additionally, many surgeons agree that most complex acetabular fractures involving the QLP can be managed through a single approach (40). To minimize the surgical trauma and complications, anterior intrapelvic approaches such as the ilioinguinal approach and the modified Stoppa approach are commonly used in clinical practice. There have been deemed to be direct accesses and reductions of fractures in the QLP and anterior column. Although several studies have reported no significant difference in perioperative complications between the two approaches, the modified Stoppa approach is associated with shorter operative time, less intraoperative bleeding and fewer neurovascular injuries (41). However, the modified Stoppa approach also has some deficiencies. Bible et al. (42) conducted a cadaveric study and showed that the modified Stoppa approach allows exposure of approximately 2 cm above and 5 cm below the pelvic brim along the QLP. Therefore, the high anterior column and most both–column fractures beyond the limits of this approach would not obtain satisfying reduction and fixation. Furthermore, the application of the partial fracture reduction tool is somewhat limited by this approach. Sagi et al. (43) reported a series of 57 patients with complex acetabular fractures using the modified Stoppa approach, but the assistant lateral window was required in 60% of the cases. Recently, Ciolli et al. (17) reported a usage rate of up to 47% for the first window of the ilioinguinal approach in their series of 34 patients with acetabular fractures involving the anterior column through the anterior intrapelvic approach (Stoppa approach). Therefore, an auxiliary approach is needed for most complex acetabular fractures when utilizing the modified Stoppa approach.

The anterior ilioinguinal approach was first developed by Letournel (7) in 1965, and it is considered an extensile approach that provides excellent visualization and exposure of the entire internal iliac fossa and pelvic brim, as well as indirect access to the quadrilateral surface. It offers three working windows that provide adequate operating angles and space, enabling the surgeon to work “down and into” the pelvis and insert quadrilateral screws from above, similar to our technique. In addition, for fractures with severe medial displacement of the QLP, adequate exposure is crucial to utilize of more reduction techniques and better application of reduction tool. Therefore, Scrivano et al. (44) suggested that compared with the modified Stoppa approach, the ilioinguinal approach was helpful to achieve a higher reduction quality of the quadrilateral fracture. Although the anterior ilioinguinal approach does not allow for the fixation of posterior column fractures, in our study, plate-screw structure fixation can be utilized in combination with the buttressing force provided by the quadrilateral screws on the surface of the QLP or together with posterior column lag screws to effectively control rotation and stabilize the fracture fragment.

It is important to note that dissection of the inguinal canal, femoral nerve, and opening the middle window of the ilioinguinal approach was necessary to access the medial surface of quadrilateral. Recent studies have shown that a higher Body Mass Index and waist-to-hip ratio are risk factors for complications related to infection and wound healing associated with surgical treatment of acetabular fractures. Avilucea et al. (45) also found that a lower muscle-to-adipose ratio at the surgical site predicted a higher risk of wound complications after acetabular fractures. In this study, one overweight patient suffered from delayed wound healing and developed an inguinal hernia, which was repaired again after the wound healed. These factors may be related. The obturator nerve could not be inspected during the approach. Sagi et al. (43) proposed that obturator nerve injury was attributed to traumatic injury and iatrogenic stretch during exposure of the QLP. Kim et al. (46) reported obturator nerve injury in 2/22 (9%), confirming that it was related to the extent of displacement of the QLP, which was not attributed to the approach. There was no intraoperative iatrogenic obturator nerve injury in the present study, partly attributed to ours experience with this approach. It is important to note that preoperative obturator nerve injury was neglected in this study. The possible reasons for this include missed diagnosis because of pain and immobilization of the limbs in the acute stage after injury or that preoperative physical examination for obturator nerve injury was not performed.

This study has some limitations. Firstly, this technique is an exceptional alternative for repairing acetabular fractures involving the QLP, including both-column fractures that mainly affecting the anterior column, transverse fractures with anterior displacement, anterior column fractures affecting the QLP, and most anterior and hemi-transverse and T-fractures, especially involving comminution and osteopenia. However, for displaced acetabular fractures mainly affecting the posterior wall or column, or for delayed surgery beyond three weeks, other approaches or fixation techniques are inevitable. Secondly, because the pelvic ring carries the transmission of mechanical force, for patients with both QLP fractures and unstable pelvic ring, restoring the stability of the pelvic ring should be *a priori*ty, the specialized contoured plate and quadrilateral screw fixation systems can provide sufficient biomechanical stability. Thirdly, the present study was a single-center retrospective study with a small number of clinical cases, and did not include a comparison between alternative surgical treatment options, which may result in selection bias. Therefore, a multicenter prospective randomized controlled trial with larger sample sizes is required to validate our findings in the future. Additionally, further hierarchical analysis is useful. Nevertheless, we believe that, if the shaping method of the plate and the nailing technique of the quadrilateral screw are executed correctly, combine the pre-contouring of the plate based on the preoperative 3D printed fracture models can further shorten the operation time, excellent repair of complicated acetabular fractures can be obtained.

## Conclusion

The management of acetabular fractures involving the QLP remains a formidable technical challenge for surgeons. Regarding the current research development direction of acetabular fracture, new surgical techniques and internal fixation devices continue to be the focus of future research in the treatment of acetabular fracture. In this study, we have introduced a novel method for reducing and stabilizing acetabular fractures involving the QLP. This technique involves using a special contoured plate and quadrilateral screws, which are inserted through the ilioinguinal approach. The procedure is uncomplicated and easily reproducible, without requiring special device. Furthermore, our case series has demonstrated that this technique provides stable and secure fixation for acetabular fractures with medial displacement, resulting in excellent radiological and functional outcomes. Therefore, we are confident that this simple and efficient technique will serve as a viable option for repairing complex acetabular fractures.

## Data Availability

The original contributions presented in the study are included in the article/Supplementary Material, further inquiries can be directed to the corresponding author.

## Supplementary material

Supplementary File 1 The process of contouring and placing the pelvic brim reconstruction plate are demonstrated.

Supplementary File 2 The process of placing quadrilateral screws as shown in the animation.

Supplementary File 3 Relevant clinical data supporting the findings of this study was provided.
